# Evaluation of the influence of kyphosis and scoliosis on intervertebral disc extrusion in French bulldogs

**DOI:** 10.1186/s12917-017-1316-9

**Published:** 2018-01-05

**Authors:** Maria Claudia C. M. Inglez de Souza, Richard Ryan, Gert ter Haar, Rowena M. A. Packer, Holger A. Volk, Steven De Decker

**Affiliations:** 1 Department of Surgery, University of São Paulo-School of Veterinary Medicine and Animal Science, Av. Prof. Dr. Orlando Marques de Paiva, 87, São Paulo, Cidade Universitária Brazil; 2 Department of Clinical Science and Services, Royal Veterinary College, University of London, Hawkshead lane, AL9 7TA North Mymms, Hatfield, UK

**Keywords:** Vertebral malformation, Hemivertebra, Intervertebral disc disease, Intervertebral disc herniation, Brachycephalic

## Abstract

**Background:**

Although thoracic vertebral malformations with kyphosis and scoliosis are often considered incidental findings on diagnostic imaging studies of screw-tailed brachycephalic breeds, they have been suggested to interfere with spinal biomechanics and intervertebral disc degeneration. It is however unknown if an abnormal spinal curvature also predisposes dogs to develop clinically relevant intervertebral disc herniations. The aim of this study was to evaluate if the occurrence of thoracic vertebral malformations, kyphosis or scoliosis would be associated with a higher prevalence of cervical or thoracolumbar intervertebral disc extrusion in French bulldogs.

**Results:**

French bulldogs that underwent computed tomography for reasons unrelated to spinal disease (*n* = 101), and French bulldogs with thoracolumbar (*n* = 47) or cervical intervertebral disc extrusion (*n* = 30) that underwent magnetic resonance imaging were included. There was a significant association between the presence of kyphosis and the occurrence of intervertebral disc extrusion, particularly in the thoracolumbar region. Dogs with kyphosis were at nearly a two times increased odds of being affected by intervertebral disc extrusion than those without kyphosis [(OR = 1.98 (95% CI: 1.04–3.78)]. There was also an association between the presence of scoliosis and the anatomical distribution of intervertebral disc extrusions, with dogs with scoliosis more likely to have more caudal lumbar intervertebral disc extrusions. Presence of scoliosis was not associated with an increased odds of being affected by intervertebral disc extrusion.

**Conclusions:**

Although thoracic vertebral malformations with kyphosis only rarely cause spinal cord dysfunction in itself, French bulldogs with kyphosis appear to be at higher risk to develop thoracolumbar intervertebral disc extrusion.

**Electronic supplementary material:**

The online version of this article (doi: 10.1186/s12917-017-1316-9) contains supplementary material, which is available to authorized users.

## Background

Thoracic congenital vertebral body malformations, including hemivertebra with kyphosis and scoliosis are frequently encountered in brachycephalic screw-tailed dogs, such as the French bulldog (FB) [1–10]. The pathophysiological mechanism resulting in development of clinical signs is considered multifactorial with vertebral misalignment, instability and vertebral stenosis considered important factors [1, 3, 11]. This condition is however most often identified as an incidental radiological finding and 78% up to 93% of neurologically normal FBs will demonstrate evidence of congenital vertebral body malformations with or without spinal kyphosis on diagnostic imaging studies of the vertebral column [3, 12, 13]. Recent studies have suggested that the severity of eventually present spinal kyphosis should be considered a critical factor in the development of spinal cord dysfunction [3, 7, 9]. Although thoracic vertebral body malformations with kyphosis is only rarely considered the primary cause of clinical signs, it has been suggested these malformations can be associated with alterations in spinal biomechanics [1, 3, 6, 7]. Changes in biomechanical properties can be associated with secondary degenerative changes of the vertebral column [6, 14, 15]. Thoracic vertebral body malformations with kyphosis have been associated with subsequent early degeneration of adjacent intervertebral discs and altered distribution of intervertebral disc extrusions (IVDEs), along the vertebral column in FB [5]. It is currently however unclear if the presence of congenital thoracic vertebral body malformations with kyphosis or scoliosis also increases the actual risk of suffering from cervical or thoracolumbar IVDE. The aim of this retrospective observational study was therefore to investigate the relationship between congenital thoracic vertebral body malformations, kyphosis, and scoliosis and the occurrence of cervical and thoracolumbar IVDE in FBs. It was hypothesized that FBs with kyphosis or scoliosis would have an increased risk to develop IVDE, would have a different anatomical distribution of IVDE along the vertebral column and would develop IVDE at a younger age when compared with those without spinal curvature abnormalities.

## Methods

### Case selection

The digital medical database of the Small Animal Referral Hospital, Royal Veterinary College was retrospectively reviewed from November 2010 to September 2016 to identify (1) a group of FBs with thoracolumbar or cervical IVDE (Hansen type I intervertebral disc disease) diagnosed by magnetic resonance imaging (MRI) [16, 17] and (2) a control group of FBs that underwent computed tomography (CT) of the thoracic vertebral column for reasons unrelated to spinal disease. For all dogs, the imaging studies and complete medical records had to be available for review. To be included in the first group, dogs had to have clinical signs and imaging findings compatible with IVDE. Dogs were excluded if the medical records or imaging studies were unavailable, if imaging studies did not include the complete thoracic vertebral column, or if any other orthopaedic or spinal disorder was detected. To be included in the second group, dogs had to be free of obvious clinical signs related to spinal or orthopaedic disease. Information retrieved from the medical records included age, gender, neutering status, clinical signs, reason for undergoing MRI or CT, final diagnosis, and initiated treatment.

### Imaging

For the group of FBs with intervertebral disc disease, thoracolumbar or cervical IVDE was diagnosed by high-field MRI under general anesthesia (1.5 T scanner; Intera; Philips Medical Systems) and included a minimum of T2-weighted (repetition time (ms) (TR)/echo time (ms) (TE); 3333/110) and T1-weighted (TR/TE, 515/15) sagittal and transverse images. Slice thickness for sagittal and transverse images were respectively 1.75 and 2.5 mm with an interslice gap of 0.3 mm in both planes. Magnetic resonance imaging included the entire thoracic vertebral column, regardless of location of IVDE.

The affected intervertebral disc space, occurrence (present or absent), number and anatomical location of thoracic vertebral body malformations, occurrence of spinal kyphosis, and scoliosis were recorded for each case. Occurrence of vertebral malformations, kyphosis and scoliosis was not recorded for the cervical vertebral column. Presence and location of IVDE was assessed on T2-weighted sagittal images, while presence, number, and location of hemivertebra and presence of kyphosis were evaluated on sagittal T1-weighted images. A thoracic vertebral body malformation was defined as any defect in vertebral body formation as outlined previously [4]. For the purpose of this study, kyphosis was defined as a dorsal spinal curvature with a Cobb angle exceeding 10 degrees. The Cobb angle was measured automatically using a commercial plug-in device as described previously [9]. When multiple hemivertebrae were present, the vertebral segment with the greatest degree of spinal angulation was chosen. Because the anatomy of the canine vertebral column is normally not characterised by any deviation in the lateral plane, presence of scoliosis was subjectively evaluated and defined as any lateral vertebral angulation on dorsal plane MRI sequences, survey ventrodorsal or dorsoventral radiographs or dorsally or 3D reconstructed CT images when available.

For the group of FBs without spinal disease, CT imaging was performed under sedation or general anesthesia for a variety of clinical indications. A 16-slice helical CT scanner, was used in all cases (PQ 500, Universal Systems, Solon, Ohio or Light Speed series, GE Healthcare, Milwaukee). The CT settings for image acquisition were: helical mode, 1–2 mm slice thickness, −1 interval between slices, 140 kVp, 120 mA, 110 mm acquisition field of view, bone and soft tissue reconstruction algorithms, 512 × 512 matrix. After completion of the axial CT study, sagittal, transversal and 3D surface reconstructions were made. Computed tomography included the entire vertebral column, regardless of the reason for clinical presentation. Occurrence, number, and location of thoracic vertebral body malformations, occurrence of spinal kyphosis and scoliosis were recorded as outlined above. Sagittal and 3D surface reconstructions were used for assessment of thoracic vertebral body malformations and spinal kyphosis. Dorsal reconstructions and 3D surface reconstruction were used for assessment of spinal scoliosis. Standard image archiving and communication system software (Osirix Foundation, V.5.5.2 Geneva, Switzerland) was used to review all cases. All imaging studies were reviewed independently by 2 observers (CI and SDD), after which a consensus agreement was reached for discordant cases. Cases for which no consensus agreement could be reached were excluded from further analysis.

### Statistical analysis

Statistical analysis of the data was performed in SPSS version 22. Pearson’s chi-squared analysis (*X*^2^) was used to detect univariate associations between the presence (1/0) of kyphosis, scoliosis or thoracic vertebral body malformations and the presence of cervical or thoracolumbar IVDE as two separate analyses. In the group of dogs diagnosed with thoracolumbar (*n* = 47) or cervical (*n* = 30) IVDE, t-tests were used to identify differences in the age of presentation with IVDE between dogs with and without spinal deformities (kyphosis, scoliosis). In addition, Pearson’s chi-squared analysis was used to identify associations between the presence of spinal deformities and the anatomical distribution of cervical and thoracolumbar IVDE. Finally, all IVDE cases (cervical and thoracolumbar) were combined (*n* = 77) and compared with controls, with Pearson’s chi-squared analysis used to detect associations between spinal deformities and IVDE presence, and t-tests used to detect differences in age between case and control dogs. Any factors found to have liberal associations with IVDE at the univariate level (*p* < 0.2) were taken forward into a multivariable binary logistic regression, using manual backwards stepwise elimination. Histograms were used to visually inspect the distribution of variables for normality, with normally distributed data presented as mean ± SD. All tests were used two-sided with *P* < 0.05 being considered statistically significant.

## Results

This study included a group of 178 FB’s, which consisted of 47 dogs with thoracolumbar IVDE, 30 with cervical IVDE and 101 FB’s underwent CT imaging for reasons unrelated to spinal disease (Table 1 and Additional file 1).

**Table 1:** Signalment and imaging findings in 178 French bulldogs with thoracolumbar IVDE (*n* = 47), cervical IVDE (*n* = 30) or without IVDE (*n* = 101)

Variable	French bulldogs with thoracolumbar IVDE (*n* = 47)	French bulldogs with cervical IVDE (*n* = 30)	French bulldogs without IVDE (*n* = 101)	Risk factor for thoracolumbar or cervical IVDE?
Male (%)	31 (66)	21 (70)	76 (75.2)	N/A
Female (%)	16 (34)	9 (30)	25 (24.8)	N/A
Mean age in years (range)	3.6 (1.6–6.6)	3.4 (1.4–6.9)	2.25 (0.33–10.2)	Yes (*p* < 0.001; OR 1.03)
Thoracic vertebral malformation (%)	44 (93.6)	24 (80)	90 (89)	No
Scoliosis (%)	13 (27.7)	6 (20)	21 (20.8)	No
Kyphosis (%)	27 (57.4)	14 (47)	33 (33)	Yes (*p* = 0.038; OR 1.98)
Median Cobb angle for kyphosis (range)	21.8 (1–47.9)	9.9 (0.6–67)	9.1 (0.2–72)	N/A

*IVDE* Intervertebral disc extrusion

### Included animals

Forty-seven dogs had thoracolumbar IVDE. This group included 31 males (23 neutered) and 16 females (11 neutered), between 1.6 and 6.6 years old (mean, 3.6 years). Presenting clinical signs included spinal hyperaesthesia without neurological deficits (*n* = 1 dog), ambulatory paraparesis with ataxia of the pelvic limbs (*n* = 17 dogs), non-ambulatory paraparesis (*n* = 17 dogs), paraplegia with intact nociception (*n* = 11) and paraplegia with absent nociception (*n* = 1 dog). The most common affected intervertebral disc spaces were T13-L1 and L2-L3 (*n* = 10 dogs for both), followed by L1-L2, L4-L5 (*n* = 7 dogs for both), L3-L4 (*n* = 6 dogs), T12- T13 (*n* = 4 dogs), T10-T11, L5-L6, and L6-L7 (*n* = one for each). Forty-four dogs (93.6%) in this group had one (*n* = 10 dogs) or more (*n* = 34 dogs) thoracic vertebral body malformations with 27 dogs (57.4%) demonstrating spinal kyphosis and 13 (27.7%) demonstrating spinal scoliosis. None of the IVDEs were located within the kyphotic or scoliotic vertebral segment. The Cobb angle for kyphosis ranged from 1 to 47.9 degrees (median 21.8 degrees) in this group of dogs.

Thirty dogs were diagnosed with cervical IVDE. This group included 21 males (11 neutered) and 9 females (7 neutered), between 1.4 and 6.9 years old (mean, 3.4 years). Presenting clinical signs included cervical hyperaesthesia without neurological deficits (*n* = 16 dogs), ambulatory tetraparesis with ataxia affecting all limbs (*n* = 12) and non-ambulatory tetraparesis (*n* = 2 dogs). The most common affected intervertebral disc spaces were C3-C4 (*n* = 16 dogs), followed by C2-C3 (*n* = 6 dogs), C4-C5 (*n* = 5 dogs), and C5-C6 (*n* = 3 dogs). Twenty-four dogs (80%) in this group had one (*n* = 5 dogs) or more (*n* = 19 dogs) thoracic vertebral body malformation with 14 dogs (47%) demonstrating spinal kyphosis and 6 (20%) demonstrating spinal scoliosis. The Cobb angle for kyphosis ranged from 0.6 to 67 degrees (median 9.9 degrees) in this group of dogs. In 12 of these dogs, orthogonal thoracic radiographs, including the complete thoracic vertebral column were additionally available for review.

One hundred and one dogs underwent a CT study, which included the thoracic vertebral column, for reasons unrelated to spinal disease. This group included 76 males (26 neutered) and 25 females (7 neutered), between 4 months and 10.2 years old (mean, 2.25 years). Reasons for undergoing CT imaging included brachycephalic obstructive airway syndrome (*n* = 88 dogs), neoplastic disease (*n* = 6 dogs), cardiac disease (*n* = 5 dogs) and gastro-intestinal disease (*n* = 2 dogs). All animals were free of apparent neurological signs. Ninety dogs (89%) in this group had one (*n* = 20 dogs) or more (*n* = 70 dogs) thoracic vertebral body malformations with 33 dogs (33%) demonstrating spinal kyphosis and 21 (20.8%) demonstrating spinal scoliosis. The Cobb angle for kyphosis ranged from 0.2 to 72 degrees (median 9.1 degrees) in this group of dogs.

### Influence of kyphosis, scoliosis and age on intervertebral disc extrusion

Compared to the control group of FBs that underwent CT for reasons unrelated to spinal disease, there was an association between the presence of kyphosis and IVDE overall (cervical and thoracolumbar IVDE combined; *X*^2^ = 7.61, *p* = 0.006). Dogs with kyphosis were more likely to be affected by cervical or thoracolumbar IVDE. Binary logistic regression revealed that dogs with kyphosis were at nearly a two times increased odds of being affected by IVDE overall than those without kyphosis [OR = 1.98 (95% CI: 1.04–3.78), df = 1, *p* = 0.038]. There was no significant association between the presence of IVDE overall and the presence of scoliosis (*p* = 0.539) or thoracic vertebral body malformations (*p* = 0.867).

**Fig. 1:**
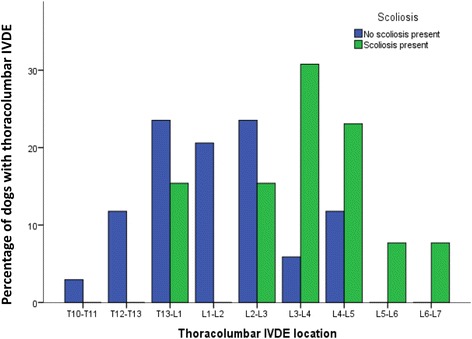
Distribution of intervertebral disc extrusions (IVDE) along the vertebral column in 47 French bulldogs with thoracolumbar IVDE. Compared to French bulldogs without scoliosis, those with scoliosis have a significantly different anatomical distribution of IVDE with the caudal lumbar disc spaces significantly more often affected

When looking exclusively at FBs with thoracolumbar IVDE, there was an association between the presence of kyphosis and presence of thoracolumbar IVDE (*X*^*2*^ = 8.17, *p* = 0.004) with kyphosis significantly more often observed in FBs with thoracolumbar IVDE compared to FBs that underwent CT imaging for reasons unrelated to spinal disease. There was no association between presence of thoracolumbar IVDE and the presence of scoliosis (*X*^*2*^ = 0.855, *p* = 0.355) or thoracic vertebral body malformations (*X*^*2*^ = 2.06, *p* = 0.151). For FBs diagnosed with thoracolumbar IVDE, there was no significant association between the presence of kyphosis (*t* = 1.03, *p* = 0.311) or scoliosis (*t* = −0.48, *p* = 0.633) and the age of development of IVDE. For FBs diagnosed with thoracolumbar IVDE, there was no significant influence of kyphosis on the anatomical distribution of IVDE along the vertebral column (*X*^2^ = 10.51, *p* = 0.231). There was however a significant association between the presence of scoliosis and anatomical distribution of thoracolumbar IVDE, with the caudal lumbar intervertebral disc spaces more likely affected in FBs with scoliosis (*X*^2^ = 15.78, *p* = 0.046) (Fig. 1).

When looking exclusively at FBs with cervical IVDE, there was no association between the presence of kyphosis (*X*^*2*^ = 1.969, *p* = 0.161), scoliosis (*X*^*2*^ = 0.009, *p* = 0.925), or thoracic vertebral body malformation (*X*^2^ = 1.699, *p* = 0.192) and presence of cervical IVDE. For FBs diagnosed with cervical IVDE, there was no significant association between the presence of kyphosis (*t* = 1.258, *p* = 0.219) or scoliosis (*t* = −0.49, *p* = 0.629) and the age of development of IVDE. There was also no significant influence of kyphosis (*X*^2^ = 0.402, *p* = 0.940) or scoliosis (*X*^2^ = 3.307, *p* = 0.347) on the anatomical distribution of IVDE along the cervical vertebral column.

Compared to the control group of FBs that underwent CT for reasons unrelated to spinal disease, dogs with cervical or thoracolumbar IVDE were significantly older (dogs with IVDE: mean ± SD = 42.36 months ±15.61, *n* = 77 vs. unaffected: mean ± SD = 26.96 months ±25.45, *n* = 101, *p* < 0.001). Binary logistic regression revealed that older dogs were more likely to be affected by cervical or thoracolumbar IVDE. Every increase in age by 1 month was associated with an odds ratio of 1.03 to be affected by cervical or thoracolumbar IVDE [OR = 1.03 (95% CI: 0.02–1.05), df = 1, p < 0.001].

## Discussion

This study evaluated the association between the presence of congenital thoracic vertebral body malformations, spinal kyphosis, spinal scoliosis and the development of clinically relevant cervical and thoracolumbar IVDE in FBs. Although an association between spinal kyphosis and adjacent intervertebral disc degeneration has been demonstrated previously [5], the results of this study suggest that spinal kyphosis should also be considered an independent risk factor for development of clinically relevant thoracolumbar IVDE. In agreement with previous findings, higher age was also demonstrated to be an independent risk factor for cervical or thoracolumbar IVDE [18]. Although IVDE is commonly encountered in FBs [6], dogs with kyphosis were at nearly twice the odds of being affected by IVDE than those without kyphosis. Additionally, our results support previous observations [6] that spinal curvature abnormalities can result in a different anatomical distribution of thoracolumbar IVDE in FBs and that disc extrusions do not usually occur in the kyphotic vertebral segments.

A short and screw-shaped tail is commonly encountered in FBs. Active breeding towards this desirable phenotypic trait is however associated with an increased number and more severe grade of thoracic hemivertebrae [10]. In agreement with previous studies [3, 4, 9, 12, 13], congenital thoracic vertebral body malformations were observed in the vast majority of neurologically normal FBs. In approximately one third of neurologically normal FBs vertebral body malformations were associated with spinal kyphosis. Although these anomalies will only rarely result in spinal cord dysfunction in itself [3, 13], the results of this study suggest that spinal kyphosis can potentially be associated with a higher risk of other spinal problems. The significantly higher number of kyphosis diagnosed in FBs with thoracolumbar IVDE, compared to those without IVDE, suggests at least an indirect link between kyphosis and the development of intervertebral disc disease.

The aetiology of canine intervertebral disc disease is considered multifactorial with genetic, biomechanical and anatomical factors involved [19]. Several studies have suggested an important role of genetic factors with a strong association between chondrodystrophy and canine intervertebral disc disease [20–22]. Chondrodystrophy is characterised by disturbed endochondral ossification, resulting in dogs with disproportionally short limbs and relative long spines [19, 23]. Although there are currently no strict criteria available to define a breed as chondrodystrophic [24], FBs are considered chondrodystrophic by some authors [5, 6, 12, 24], while they are not considered chondrodystrophic by others [20]. It is therefore possible that the high prevalence of IVDE in FBs can, at least in part, be explained by their chondrodystrophic or chondrodysplastic phenotype. Heritability of spinal curvature abnormalities and a genetic relationship between spinal curvature and other spinal conditions, including intervertebral disc degeneration, has been considered and evaluated in people [25, 26]. Although the occurrence of hemivertebra is considered heritable in FBs [10], it is currently unclear if a genetic relationship between spinal kyphosis and intervertebral disc degeneration exists in this specific breed. It should further be noted that thoracic vertebral malformations and spinal curvature abnormalities occur also in other brachycephalic ‘screw-tailed’ breeds, such as Pugs and English bulldogs [2, 4, 13]. While the French bulldog is listed among the most common breeds affected by IVDE [6, 24], this is not the case for Pugs and English bulldogs. This suggests that the occurrence of spinal curvature abnormalities cannot solely explain the high prevalence of IVDE in French bulldogs and should rather be considered an additional risk factor for development of this disorder.

Spinal curvature abnormalities have been suggested to result in vertebral instability and altered biomechanical loading of the vertebral column [3, 6]. Both biomechanical overloading and immobilisation have the potential to result in accelerated intervertebral disc degeneration [14]. Changes in the local mechanical environment and nutritional supply have been suggested to cause early intervertebral disc degeneration in the affected vertebral segments of dogs with kyphosis and people with scoliosis [5, 14]. In agreement with the findings of Aikawa [6], IVDE did not occur in the thoracic kyphotic vertebral segments, but in the more caudal thoracolumbar and lumbar intervertebral disc spaces. Because of the stabilising and protective effects of the intercapital ligament, herniation of the intervertebral discs cranial to T10 should however be considered rare in dogs overall [24]. Kyphotic vertebral segments have however been suggested to result in altered biomechanical stress over the entire vertebral column [6] and it can therefore be assumed that thoracic kyphosis is associated with abnormal biomechanical loading of the more distant thoracolumbar and lumbar intervertebral discs. Similarly, people with thoracic scoliosis, the most common spinal curvature abnormality in humans, demonstrate altered biomechanics of the lower lumbar spine [27] and are at higher risk of recurrence of symptoms after caudal lumbar disc surgery [28]. In the study presented here, FBs with scoliosis had a different anatomical distribution of thoracolumbar IVDE along the vertebral column. More specifically, dogs with scoliosis had IVDE affecting the caudal lumbar disc spaces more often, compared to FBs without scoliosis (Fig. 1). Although this finding supports the hypothesis that kyphosis and scoliosis are associated with altered biomechanical loading along the entire vertebral column, further studies are necessary to objectively evaluate the biomechanical consequences of spinal curvature abnormalities in dogs.

Considering the factors above, it is possible that FBs are at risk of thoracolumbar IVDE because of genetic factors and that biomechanical factors associated with spinal kyphosis should be considered an additional risk factor for developing thoracolumbar IVDE.

Although development of clinical signs as a direct consequence of thoracic vertebral body malformations is considered multifactorial, the degree of spinal kyphosis has been suggested to be a key factor. Cobb angles exceeding 35 degrees have been associated with a high probability of developing neurological deficits [9]. Several neurologically normal FBs and FBs with thoracolumbar or cervical IVDE displayed kyphosis with Cobb angles exceeding 35 degrees (up to 72 degrees). Although it cannot be excluded that these dogs demonstrated subtle gait abnormalities as a consequence of their kyphosis, this was never appreciated by their owners or observed by the responsible clinicians. This finding also illustrates that the presence of severe kyphosis is not necessarily associated with obvious signs of spinal cord dysfunction and that even in dogs with radiographic evidence of severe spinal curvature abnormalities considerations should be given to other causes of spinal cord dysfunction. Although it can be hypothesized that higher degrees of spinal kyphosis can be associated with more pronounced alterations in vertebral biomechanical loading, further studies are necessary to evaluate if a higher degree of kyphosis is indeed associated with a higher risk of developing thoracolumbar IVDE.

This study is limited by several factors. Although the group of control dogs did not have any reported or observed gait abnormalities, the majority of these dogs did not receive a complete neurological examination. It can therefore not be excluded that some dogs could have demonstrated subtle gait abnormalities associated with a thoracic vertebral body malformation or kyphosis. Similarly, although the clinical presentation of FBs with IVDE was always compatible with the imaging findings of thoracolumbar or cervical IVDE, it cannot be excluded that these dogs demonstrated pre-existing subtle gait abnormalities associated with their vertebral anomalies. Further, it cannot be excluded that some dogs that underwent CT for reasons unrelated to spinal disease would have developed cervical or thoracolumbar IVDE later in life. This is especially true because French bulldogs in the control population were significantly younger than the dogs affected by IVDE. Binary logistic regression however indicated that higher age and occurrence of spinal kyphosis should both be considered independent and non-related risk factors for occurrence of IVDE in FBs. It can also not be excluded that some dogs would have developed more severe degrees of kyphosis later in life [29, 30]. Control dogs underwent CT, while dogs with IVDE underwent MRI as the most appropriate diagnostic technique for their respective disorders. Although easy to recognise and well-defined diagnostic criteria were used to recognise thoracic vertebral body malformations, spinal kyphosis and spinal scoliosis, little is known on how both imaging modalities compare for these specific purposes. Several studies in human medicine have compared radiography vs. CT [31, 32] and radiography vs. MRI [33, 34] to evaluate and quantify spinal curvature abnormalities, while only a few studies have compared all three imaging techniques for this purpose [35, 36]. The results of these studies indicate that CT and MRI can be used to evaluate and quantify spinal curvature abnormalities with a good reliability, correlation and agreement among the three imaging techniques. Because dogs with IVDE underwent MRI and control dogs underwent CT, reviewers were not blinded to the clinical status of each dog. Although images were reviewed independently by two observers after which a consensus opinion was reached, it cannot be excluded that this has influenced image interpretation. Finally, the complete vertebral column was not included in most MRI studies and CT studies. It can therefore not be excluded that radiographic signs of IVDE could have been present, even in the absence of obvious clinical signs.

## Conclusion

Although thoracic hemivertebra with kyphosis does only rarely cause spinal cord dysfunction in itself, FBs with kyphosis appear to be at higher risk of developing IVDE overall and thoracolumbar IVDE in particular. Spinal scoliosis is further associated with an altered anatomical distribution of IVDE, with FBs with scoliosis suffering more likely from caudal lumbar IVDE. Further studies are necessary to objectively evaluate the biomechanical consequences of spinal kyphosis and scoliosis in dogs.

## Additional file

Additional file 1: Demographic data, presence of intervertebral disc extrusion, hemivertebra, kyphosis, scoliosis and Cobb angle of 178 French bulldogs with our without intervertebral disc extrusion. (XLSX 16 kb)

## Abbreviations

CT: Computed tomography
FB: French Bulldog
IVDE: Intervertebral disc extrusion
MRI: Magnetic resonance imaging
